
JOURNAL INFORMATION
==============================
NLM Title Abbreviation: J Can Health Libr Assoc
Journal ID: J Can Health Libr Assoc
NLM Journal ID: 101229936
Journal ID: JCHLA

The Journal of the Canadian Health Libraries Association

EISSN: 1708-6892
Publisher: Canadian Health Libraries Association / Association des bibliotèques de la santé du Canada

ARTICLE INFORMATION
==============================
PMCID: PMC12352438
DOI: 10.29173/jchla29897
Article ID: JCHLA-46-053
Article version: 1
Subjects: Abstracts

CHLA 2025 CONFERENCE CONTRIBUTED PAPERS / ABSC CONGRÈS 2025 COMMUNICATIONS LIBRES

Electronic publication date: 2025 Aug 1
Publication date: 2025 Aug
Volume: 46
Issue: 2
First page: 53
Last page: 62
Copyright: © Fournier
Copyright year: 2025
License: This article is distributed under a Creative Commons Attribution License: https://creativecommons.org/licenses/by/4.0/
License URL: https://creativecommons.org/licenses/by/4.0/

==============================

## CP1. A scoping review case study of citation searching indexes and tools

Colleen Pawliuk
BC Children's Hospital

**Background:** Citation searching is a valuable form of supplementary searching for scoping reviews, but is often time consuming. Several citation indexes and tools are now available to make this process more efficient, but it is unclear which may provide the best return on investment. Past studies have investigated the value of citation indexes in the context of systematic reviews or the coverage provided. However, these studies are limited in value as they do not include a robust grey literature search, do not include citation searching tools (e.g. CitationChaser), or do not quantify the value of the indexes with the number of relevant studies identified. **Objective:** To test citation searching indexes/tools in the context of a scoping review that includes grey literature to quantify value of each index/tool. **Methods:** A literature search was conducted to find citation searching indexes/tools that allow for bulk download of references. All citation indexes/tools were used for backward and forward citation searching in the scoping review. Recorded for each tool was: time needed to complete citation searching, total number of references retrieved and number of relevant references retrieved. Sensitivity and precision of backward and forward citation searching will be calculated for each tool using any relevant citations identified through citation searching as a reference set. **Results:** Descriptive statistics for each tool will be shared along with recommendations for which tool(s) may provide the best balance of time spent and relevant references found.

## CP2. A scoping review of automated indexing in Medline -- how did we get here?

**Background:** Medline's 2022 transition to automated indexing has had a profound impact on the precision, accuracy and completeness of MeSH headings applied to records, and consequently, on the findability and usability of publications. Health science librarians must adapt accordingly. We will document the rise of automated indexing in Medline, and briefly touch on its present-day growing pains. **Methods**: We searched the usual places: MEDLINE and EMBASE (both via Ovid), the Web of Science Core Collection, CINAHL and LISA (Library and Information Science Abstracts; both via EBSCO), and went on to hand-search key journals, track citations, and peruse a wide range of grey literature. We are guided by Arksey and O'Malley's 2005 framework, with updates from Levac et al (2010) and the 2020 JBI Guidelines. Our protocol was deposited on the Open Science Framework (OSF) in November 2024 (https://osf.io/g4q8u/). Aggregated data will be thematically coded and presented as conceptual maps. We anticipate the impetus, benefits, and shortcomings of automated indexing as themes. **Results:** We will present a chronology of the development and deployment of automated indexing vis-a-vis Medline. We will make recommendations for future research on the impact of automated indexing in Medline and other health science databases, and discuss gaps identified in our review of publications. **Discussion:** Beyond the history of automated indexing, we will explore its impacts on the accuracy and completeness of Medline records, and the downstream impacts on librarian practices.

## CP3. AI research assistants and Cochrane reviews: a comparative analysis of scientific summaries

Catherine Boden, Erin Langman
University of Saskatchewan

**Introduction:** There has been a recent explosion of AI-powered research assistants designed to find and summarize scientific literature. These nascent tools require evaluation to foster critical thinking about their use and to inform improvements. This study compares AI-generated syntheses of the literature to the conclusions of Cochrane systematic reviews of the same clinical questions. **Methods:** Cochrane systematic reviews (SRs) of exercise with published March 2024 to 2025 were selected.  Four AI-powered research assistants (AI tools) and Copilot were queried using the SR research questions. The following data from the SRs and the AI tool summaries was extracted independently by the two researchers, with disagreements resolved through consensus: the number, study design, and citations of included articles, assessment of quality or risk of bias (if available), and conclusions regarding the intervention(s).  Agreement between SRs and AI tools will be assessed by coding conclusions as agreeing, disagreeing, or unclear. Descriptive statistics will be used to compare the literature cite for the conclusion or summary. **Results:** Data analysis is underway. **Discussion:** This analysis will inform our understanding of the accuracy, reliability and reproducibility of these AI research assistant tools.

## CP4. Anything you can do, AI can do better... or can it? Comparing ChatGPT's search strategy outputs with Cochrane review searches

Katherine Howell, Rebecca Carlson, Emily Jones, Elizabeth Moreton
University of North Carolina

**Objective:** Previous studies have measured ChatGPT's capabilities for completing literature search tasks. This study seeks to assess ChatGPT's capability to produce comprehensive search strategies for systematic reviews, specifically comparing AI-generated outputs against published Cochrane review searches for precision and recall.  **Methods:** We created a test set of 9 PubMed search strategies from recent Cochrane. A script was created and ChatGPT was queried using each Cochrane review topic, research question(s), and inclusion criteria to generate a relevant PubMed search strategy. Precision and recall were measured using the Cochrane reviews' PubMed search strategies and included articles as the standard and ChatGPT searches were evaluated using PRESS. **Results:** GenAI search strategies had lower recall and lower precision on average when compared to Cochrane search strategies. The GenAI search strategies had an average recall of 57.6% (ranging from 0% to 100%) and an average precision of 1.51% (ranging from 0% to 4.17%), while the Cochrane search strategies had an average recall of 93.7% and an average precision of 2.39%. PRESS evaluations revealed errors including hallucinated MeSH terms and issues with keywords. The results indicate that ChatGPT could be used to help develop comprehensive literature search strategies for systematic reviews, but not without librarian oversight. **Conclusion:** Results of this project provide a current estimation of whether, and to what extent, ChatGPT could be used to develop literature search strategies for systematic reviews. This project adds to the literature on GenAI uses for systematic reviews and informs librarians of the potential of these tools for comprehensive literature search development.

## CP5. Artificial intelligence-supported screening: does it impact evidence certainty and time to complete a rapid review?

Sarah Neil-Sztramko[12], Robyn Traynor[2], Emily Clark[2], Elaine Toomey[3], Maureen Dobbins[12]
[1]McMaster University, [2]National Collaborating Centre for Methods and Tools, [3]University of Galway

**Introduction:** The National Collaborating Centre for Methods and Tools (NCCMT) established its Rapid Evidence Service (RES) to support evidence-informed public health by conducting timely, rapid reviews on priority topics. Artificial intelligence (AI) screening features offer the potential to automate and expedite the review process, while reducing unintended human biases or errors, but there is limited evidence to quantify this impact. This study aims to evaluate how AI compares to manual screening with respect to missed studies, impact on overall review findings, and time to complete. **Methods:** Two AI features, Re-Rank and Check Screening Errors, were compared to manual dual screening during title and abstract screening (DistillerSR, v2.35). As screening occurred in each review, project clones were made at likelihood thresholds of 60-95%, AI screened remaining references, and potential false excludes were identified. These AI-screened results were compared with manual screening in the original projects to identify how many studies would have been missed at each threshold. The impact of omitting missed studies on the review's key findings was assessed. Finally, time spent screening was tracked across reviews. **Results:** Six rapid reviews were conducted during the study period. In preliminary data analysis, AI correctly excluded up to 60% (2600 out of 4100 studies) at a prediction threshold as low as 80% in one review; time spent screening was 47 hours. **Discussion:** AI is a promising support tool for improving screening efficiency and accuracy. Additional study is needed to understand how AI can be most appropriately integrated into rapid review methods.

## CP6. Assessing the Canadian digital health landscape: opportunities for improved data sharing and research

Alyssa Foote
World Data System

**Introduction:** The Canadian digital health landscape is a complex ecosystem with extensive data crucial for research and innovation. This analysis provides an overview of key national and provincial organizations in health data stewardship, mapping their roles, interconnections, and essential data repository attributes to highlight current health data management practices in Canada. **Method:** Identified through prior knowledge, re3data.org, and Google searches, a selection of Canadian health data entities was examined for description, users, partners, data accessibility, and re3data presence. Data repositories were further analyzed against key attributes drawn from the Research Data Alliance's "Common Descriptive Attributes of Research Data Repositories" report. **Discussion:** This analysis demonstrates that the diverse Canadian health data ecosystem exhibits varying maturity levels in data management and access. Significant variations were observed in accessibility, data catalogues, metadata standards, use of a persistent identifier (PID) system, and re3data entries, indicating inconsistencies in discoverability and transparency. **Recommendations:** To maximize the impact and FAIR (Findable, Accessible, Interoperable, Reusable) use of Canadian health data, this report recommends enhancing data management practices, including improved metadata standards, increased PID system adoption, and widespread data repository certification. The World Data System can support this through knowledge sharing and advocating for international standards. **Conclusion:** The Canadian digital health data landscape shows strengths in research and collaboration, but needs to improve in other areas such as standardization and metadata. Addressing these issues with World Data System support is crucial for a more robust and interoperable infrastructure for effective health research, benefiting all Canadians.

## CP7. Critiquing systematic review search methods in the Cass Review: developing a comprehensive checklist

Elizabeth Sanders[1], Elizabeth Yates[2]
[1]Lamar University, [2]Brock University

**Introduction:** In April 2024, the Cass Review released its final report regarding gender identity services for children and youth in the U.K. The report, based partly on the outcomes of six systematic reviews, has been used to justify restrictions on a wide swath of gender-affirming health care services and has been broadly criticized both by clinicians and 2S&LGBTQ+ advocates. As librarians striving to advance best practices in evidence synthesis research and evidence-informed care, and as individuals invested in promoting social justice, we wanted to bring our own critical lens to the Cass systematic reviews by critiquing their search strategies and related processes. **Methods:** We began by examining the literature and reviewing existing tools and approaches to critically appraise evidence synthesis reviews and/or search strategies. We then developed a new checklist incorporating elements of key resources such as PRESS, MECCIR, AMSTAR 2, and guidance created by and for librarians. We each independently created a draft checklist and then collaborated on multiple iterations before completing a version which we will pilot test in Winter 2025 and then finalize. **Results:** As of June 2025, we will share the results of using our tool to critically appraise the search methods from at least one Cass systematic review. **Discussion:** While this new appraisal tool is primarily focused on searches within the Cass publications, we hope it can be applied more broadly to other evidence synthesis reviews, particularly those impacting other vulnerable populations.

## CP8. Developing a scaffolded learning framework for librarians new to health sciences

**Background:** In early 2024, the idea to convene a group to harness Association of Academic Health Sciences Libraries (AAHSL) members' collective efforts to train and onboard librarians emerged during an AAHSL eSalon meeting. A task force came together through follow-up emails among those who expressed interest in this topic. **Description:** The task force's goals were to explore, evaluate, and recommend strategies for establishing a program to onboard and educate individuals new to health sciences librarianship efficiently. This initiative seeks to enhance the transition process for newcomers, ensure they receive comprehensive training and support to excel in health sciences libraries and ease the burden of individual institutions through shared practices and training materials. **Results/Outcomes:** Since forming the task force, we have completed a literature review, explored cohort models for new librarians, conducted an environmental scan, and begun developing a scaffolded learning framework to support the onboarding and education of new health science librarians. The task force shared the framework at the annual AAHSL meeting (November 2024) for review and feedback. This initiative, which began at a monthly meeting, seeks to prepare librarians for success in health sciences. This session will share lessons learned through the process and review the framework. Library managers and mentors can use the results of this work as a guide for training new health sciences librarians.

## CP9. Evaluating the performance of broad and narrow search strategies when using machine learning-based software for title/abstract screening

Michelle Swab
Memorial University

**Introduction:** A number of machine learning tools have been developed to enhance the efficiency of title/abstract screening in review projects. While guidance articles suggest these tools make broader, high-yield searches more feasible by saving time, the actual performance of sensitive (broad) versus precise (narrow) search strategies in this context remains under explored. **Methods:** Using ASReview, an open-source systematic review tool, I evaluated search strategy performance in a sample of completed reviews. For each, one database search was selected and revised to broaden (n = 9) or narrow (n = 1) its scope. Search results were labeled as relevant or irrelevant based on each review's included articles. These labeled sets were uploaded into ASReview's simulation module. Performance was assessed using metrics such as time to achieve true recall at 0.95. **Results:** To reach a true recall of ≥0.95, broader searches increased screening time by 10–875%, with a median increase of 25%. Systematic reviews had a smaller median increase (18%) compared to other review types (374%). At a rate of one record per minute, the added time to reach this level of recall ranged from 0.4 to 35.1 hours (median: 1.8 hours). In two cases, machine learning screening with broader searches took longer than the full manual screening of the narrower result set. **Discussion:** These findings suggest efficiencies associated with machine learning tools do not always offset the extra screening time required by broader searches. In this case study, efficiency losses were most notable in non-systematic and non-quantitative reviews.

## CP10. Investigating the impact of the NLM automatic indexer on information retrieval using citation metadata

Emma S Garlock[1], Joan C Bartlett[2]
[1]University of Ottawa, [2]McGill University

**Introduction:** As the implementation of automatic indexing for MeSH terms becomes more well-known, concerns are being raised in the field of health sciences librarianship on how these changes impact established searching practices. While other ongoing research investigates the accuracy and reliability of automatic indexing on a per-citation basis, this work analyzes overall trends and performance of the algorithm for chemistry and genetics research. **Methods:** 4302 citations published between November 2020 and March 2023 had their relevant information fields extracted via NLM's efetch and xtract tools on July 25th, 2024. This data was combined with MeSH data downloaded from the Ontology Lookup Service on September 4th, 2024. All analyses were completed in R. To evaluate the potential impact of stemming on term overlap, Porter Stemming was applied using the Tokenizer and SnowballC packages. **Results:** Results generally support the claim that automatic indexing decreases the time required for a citation to receive indexing. There is variability in how well search fields overlap for indexing methods, but overall topic harmonization increases as terms are tokenized and stemmed. Manually indexed citations tend to have a higher degree of field overlap, which aligns with the finding that the average number of MeSH terms is higher for manually indexed citations. **Discussion:** This work builds on feedback from previous presentations and provides a more detailed and large-scale investigation into the impact of the automatic indexing algorithm and its impact on health librarianship.

## CP11. Invisible in the index: how Medline indexing excludes intersex people

Eleni Philippopoulos
McGill University

**Introduction:** Since April 2022, indexing in Medline has been done through automatic indexing using an algorithm. According to PubMed, quality assurance processes are in place to ensure the accuracy of the decisions, including citations with recognized ambiguities. Humans may still participate in the indexing process by reviewing and curating the results. **Objective:** Despite the introduction of the MeSH term Intersex Persons in 2020, as of January 2025, only 13 articles are tagged with the term. None of these articles were indexed using automatic indexing. This paper examines the systemic exclusion of intersex-related literature within the indexing framework in Medline. **Methods:** In July 2024, a search was conducted to identify citations that included intersex in the title and abstract. The search was limited to articles published from 2020 onwards and excluded the 13 articles with the Intersex Person MeSH term. **Results:** A total of 500 articles were identified. After excluding animal studies and studies that were not yet indexed, 228 articles remained. A definitive decision about the indexing could not be made for 24 articles. Of the 228 articles, 167 (73.2%) were indexed using automatic indexing and the remaining 61 (26.8%) involved humans. After analyzing the full-text, it was determined that 59.9% of the automatic indexed articles and 45.9% of the manually indexed articles should have been tagged with the Intersex Persons MeSH term. **Conclusion:** This study reveals significant gaps in the Medline indexing process concerning intersex-related literature. These findings indicate systemic issues in both automatic and manual indexing processes, suggesting that intersex-related research is often misunderstood, misclassified and invisible.

## CP12. Making the case for play in your library

Glyneva Bradley-Ridout
University of Toronto

**Introduction:** Health sciences libraries serve a diverse population of adult users, providing access to varied services, supports, and spaces. There are many factors that may make a user's visit to the library a productive and joy-filled experience. In addition to the standard (and important) functions as a space for learning, study, and research, this presentation will argue that health sciences libraries are a logical space for play and fun to occur as well. **Description:** This presentation will be divided into two parts. The first part will introduce the case for play and why bringing play into library spaces is worth the time and resources. A variety of arguments to this case will be shared, included the supporting literature behind play in adult psychology, and the argument that libraries are an ideal space for this to occur. The second part of the presentation will share resources and strategies for implementing play-based activities, including utilizing strategic plans and setting budgets. A few examples of easy to execute play-based activities will be shared. **Outcomes and Conclusion:** This session will be of interest to those who wish to bring play into their library spaces, or those who would like to know more about this means. By the end of the session, attendees will have had the opportunity to reflect on the meaning of play in library settings, as well as consider applications in their own library spaces.

## CP13. Optimizing communication and data collection for a systematic review team using Microsoft Power Automate®

Emily Jones, Carrie Baldwin-SoRelle, Rebecca Carlson
University of North Carolina

**Background:** Libraries with systematic review (SR) services track and collect data on requests to manage workload and to make administrative decisions like hiring or acquiring resources based on demand. Librarians rely on technology, often selected based on institutional subscriptions, for internal tracking, communication, and data collection. However, many libraries rely on manual data entry despite available low-code software like Microsoft Power Automate or Zapier that could automate and optimize team workflows. This case study describes how a SR coordinator used Power Automate flows to automate email reminders, centralize workflows, collect data, and ensure requests were claimed by librarians across a large team. **Description:** We created a Power Automate workflow to automatically email our team of a new request upon submission. This information is transferred to our tracking system, Microsoft Lists, which is embedded into our Teams site for convenience. Librarians can claim requests and add tags, notes, or files. Finally, the form submission updates a backup Excel file we use for statistics and visualizations. These processes ensure information is centralized and automated, so team members do not have to locate or update information manually. **Conclusion:** We demonstrate how to optimize and integrate existing tools using low-code software. This strategy is not exclusive to Microsoft and is transferable to Google or other major office management software. Additional integrations including Planner are available for those preferring Kanban-style tools.

### CP14. Promoting the use of synthesized evidence in public health decision making: a rapid evidence service and repository of public health evidence syntheses

Sarah Neil-Sztramko[12], Alanna Miller[2], Robyn Traynor[2], Sophia Caldwell[2], Krishian Camargo[2], Emily Clark[2], Kristin Rogers[2], Maureen Dobbins[12]
[1]McMaster University, [2]National Collaborating Centre for Methods and Tools

**Introduction:** Evidence syntheses can be used to inform public health policy and programming decisions, however, organizations may lack the resources and expertise to develop timely, high-quality syntheses. The National Collaborating Centre for Methods and Tools (NCCMT) launched a Rapid Evidence Service (RES) to answer priority public health questions through rapid reviews. The NCCMT also established the Repository of Public Health Evidence Syntheses (the Repository) to minimize duplication of efforts, facilitate collaboration, and ensure public health practitioners have access to relevant evidence syntheses. **Description:** The RES process involves collaboratively developing a research question, conducting a comprehensive search, and critically appraising and synthesizing the findings into an actionable report. Each RES report is included in the Repository and made available to the public health community. Other organizations also contribute their syntheses to the Repository. Users can search the Repository for existing syntheses and adapt the findings for use in their own communities or connect with authors to collaboratively address public health issues. **Outcomes:** Since launching in 2020, the RES and Repository have focused on many timely public health topics, such as COVID-19, climate change, and health equity. The RES has collaborated with over 20 organizations to complete more than 100 reviews and review updates on 61 topics. The Repository hosts over 600 submissions from over 75 organizations and has been accessed over 30,000 times. **Discussion:** The RES and Repository facilitate access to relevant, high-quality evidence syntheses. These services promote the use of synthesized evidence, encourage interorganizational collaboration and ease knowledge exchange in public health decision making.

### CP15. Rooted in competence: using the MLA professional competencies to redesign a health information course

Colleen Pawliuk
BC Children's Hospital

**Background:** Health information courses in Library and Information Science programs are one pathway into health librarianship. They may raise awareness of the profession among students and provide an avenue to learn essential skills of health information practice. For those unfamiliar with health information practice, understanding how to connect course topics to marketable skills can be a challenge. **Description:** The health information course at the University of British Columbia was redesigned using the Medical Library Association (MLA) Competencies for Lifelong Learning and Professional Success as a framework. An introduction to the course was provided on the first day of class, which linked the Professional Competencies to the course topics and learning objectives. Students self-assessed their level of Professional Competency at the start and end of the course through an anonymous online survey. **Outcomes:** Overall Professional Competency scores increased for the class. Informal feedback on the course was positive and focused on the practicality of the course. **Conclusion:** Explicitly anchoring course content in the MLA Competencies can provide a way for learners self-evaluate their learning and provides a clear framework for understanding the key competencies for health information professionals.

## CP16. Search strategies as research data: new perspectives on documentation and sharing practices

Heather Cunningham[1], Julia Martyniuk[1], Jill Boruff[2], Sabine Calleja[2], Alisa Rod[2], Ani Orchanian-Cheff[3], Alix Pincivy[4], Daniela Ziegler[5], Karly Gunson[1]
[1] University of Toronto, [2]McGill University, [3]University Health Network, [4]Centre Hospitalier Universitaire Sainte-Justine, [5]Université de Montréal

**Objective:** By viewing knowledge synthesis (KS) searches as code and depositing them into data repositories, librarians assert their intellectual control over their work, ensuring that the search strategies are properly reported and presented as stand-alone intellectual outputs. To better understand how to support these initiatives, the authors investigated Canadian health sciences librarians' attitudes and behaviours regarding the documentation and sharing of KS search strategies. **Methods:** We invited 498 people to a bilingual 15-minute survey if they were listed as a health sciences librarian or information specialist on public websites of academic, hospital, government, or special libraries in Canada. **Results:** 128 complete responses were received for a 25.7% response rate. 84% of respondents agreed that search strategies and their related output files are equivalent to research data and code for a KS publication, but only about 30% have deposited search strategies in a data or institutional repository. 85% have used or adapted an existing search strategy in the creation of a new strategy. The results also show that intellectual control of co-authored search strategies is salient among participants and that there is broad interest in integrating research data management (RDM) best practices into KS work. **Conclusions:** The results suggest that Canadian librarians recognize their work as independent data; however, there are no formal guidelines to ensure that librarians integrate search deposits into their KS workflows. Our findings can be used to advance the open sharing of search strategies among Canadian librarians, aligning with PRISMA-S, and encourage librarians' continuous engagement as participants in the RDM ecosystem.

## CP17. Stronger together: adventures in instruction and resource sharing to cultivate aspiring nursing students

Marilia Antunez[1], Christa Taylor[2]
[1]University of Akron, [2]Barberton High School

**Introduction:** Literature on career technical education (CTE) programs introducing nursing research to high school students is scarce, and fewer articles discuss the involvement of librarians. This paper examines the integration of research into the curriculum of a two-year CTE program, the Advancement to Nursing (ATN). ATN is a program for high school students considering nursing or another health career. This program partners with different types of libraries, including the University Akron (UA) Libraries. As part of the ATN program, Barberton High School partnered with UA. Over the past two years, the librarian and the nursing instructor worked closely to plan the library sessions whose objectives were to improve students' database searching skills, to locate appropriate resources, and to cite sources correctly. **Description:** At UA, the librarian provided two instruction sessions annually. Students were introduced to searching strategies, peer-reviewed articles, and the American Psychological Association (APA) Style. Students searched the CINAHL database and other quality resources. An online guide was created to supplement instruction to help students with the assignments. Outcomes: A rubric was used to determine students' ability to utilize approaches introduced in library sessions, understanding of APA Style, and writing ability. Although students were able to navigate databases more effectively, they needed more assistance with citing resources. **Discussion:** The partnership improved students' ability to find appropriate information, provided assistance to help students complete their assignments, and highlighted the importance of research in nursing/health fields. Weaknesses included technological challenges and time constraints. Future plans are to increase APA Style education and writing labs.

## CP18. The Book Nook: creating a leisure reading space for staff and learners at three hospital library sites

Ashley Farrell, Bianna Glaizer, Cynthia Chui, Rouhi Fazelzad
University Health Network

**Introduction:** Existing literature highlights the benefits of leisure reading collections in academic libraries for students, such as stress reduction and improved well-being. Initiatives in hospital libraries, particularly those targeting staff, remain underexplored. The University Health Network Library implemented "The Book Nook" collection across its libraries to provide staff with access to recreational reading materials. This initiative helps to create an inviting multi-use library space, aims to promote the library's role as a hub for both clinical and leisure resources, and to contribute to staff well-being. This program will provide insight into creating, administering and evaluating a leisure collection specific to hospital staff. **Description:** The leisure reading collection at the Toronto General Hospital began after the library received a collection of donated books for its staff. In 2024, a library committee took an active role in managing the collection and worked to reorganize, rebrand, and promote the materials to improve access and awareness. The resources include a diverse selection of books, and it is run on a donation-driven model. This initiative has since expanded to two other sites. **Outcomes:** Results of a survey evaluating the impact of the collection, who is using it, and user satisfaction will be shared during the presentation. **Discussion:** This initiative highlights the potential of leisure reading programs in hospital libraries for staff and learners. Preliminary results will provide information on lessons learned developing and implementing the collection and will outline patron feedback, which will guide future improvements to the collection. Future directions include expanding this program to a fourth site.

## CP19. The development of a freely available module series introducing researchers to all stages of the systematic review process

Sandra McKeown[1], Jennifer Ritonja[23], Eleftherios Soleas[1]
[1]Queen's University, [2]McGill University, [3]St. Mary's Research Centre

**Introduction:** Many libraries continue to grapple with the growing demand for systematic review support and how to address gaps in researcher knowledge. Our previously reported survey results of Ontario medical schools and affiliated hospitals found that all 13 library respondents would find free online modules useful for supporting researchers conducting systematic reviews. **Description:** Our interprofessional team developed a module series that introduces all stages of the systematic review process. The module series is accredited for continuing professional development by the Royal College of Physicians and Surgeons Canada and the College of Family Physicians Canada. **Outcomes:** Individual modules have been posted online via Queen's University Library as they are created and links to the modules have been shared with the health sciences library community via listservs. Researchers at Queen's who contact the library for systematic review support are referred to the content, and all students are required to complete the module on searching and submit proof of a knowledge check task before meeting with a librarian. While usage statistics for the modules have been high, it's apparent during librarian consultations that many researchers are still unclear of the process and that students may not be doing the required module on searching. **Discussion:** Modules cannot replace the value that direct support from librarians, biostatisticians, or methodology experts can provide, however, they may help with addressing researchers' gaps in the systematic review process and offer libraries and institutions a more efficient way of familiarizing researchers with best practices for performing a high-quality review.

## CP20. The involvement of librarians and library technicians in knowledge syntheses published by researchers from Quebec universities: an overview

**Introduction:** This study analyzes librarians and library technicians' involvement in academic knowledge syntheses (KS) publications in the Province of Quebec. **Methods:** Using Scopus, Web of Science Core Collection, MEDLINE, LiSSa, Érudit and Google Scholar, all KS articles published between 2020 and 2024 by researchers affiliated (first or last author) to a Quebec university was collected. Screening and data extraction were conducted with Covidence. Analyses will be performed on the involvement of library workers according to types of KS, disciplines, institutions, language of the publications and affiliation to a French or English language institution. **Results:** Library workers' involvement in KS is acknowledged in various ways. Preliminary analysis (2023-2024) shows that library technicians or library teams are occasionally mentioned in the acknowledgements section. Librarians or information specialists are co-authors (30%), mentioned in the acknowledgements section (40%) or cited anonymously as consultants in the methodology section (33%). More than one librarian may contribute to a single KS publication, however, the role as a peer reviewer is rarely specified. Few KS publications were in French, and librarian co-authorship was less common in these compared to English-language publications. **Discussion:** Further analyses will explore more in-depth library worker involvement according to KS type, academic discipline, institutional affiliation, language of publication and institutions of the authors.

## CP21. Where did the reviews go?: the good, the bad, and the unfinished

Zachary Osborne[12], Carolyn Ziegler[1], Teruko Kishibe[1], Talin Boghosian[1]
[1]Unity Health Toronto, [2]Seneca Polytechnic

**Introduction:** Information specialists who collaborate on knowledge synthesis (KS) projects invest tremendous time and intellectual labour on reviews. Despite their critical role in ensuring review quality, the outcomes of their contributions—such as successful publication—remain unclear. Consequently, there is a need to analyze longitudinal data on the outcomes of collaborations such as the rate of publication success versus the number of unfinished reviews. Our study aims to understand what happens after the intense work of evidence retrieval has been completed. **Methods:** Between 2013 and 2023, information specialists from a multi-site health network collaborated with research teams on 596 KS projects. In 2024, the library team collected and analyzed KS project data from its data analytics platform, LibInsight (Springshare), with the aim of detecting trends, measuring the impact of interventions to reduce research waste, and of making recommendations for future practice. This process involved identifying the numbers of dropped projects and successful publications, of librarian co-authorships or acknowledgements, the types of review methodologies used, and more. **Results:** Preliminary findings suggest a substantial number of KS collaborations with information specialists did not result in published manuscripts. The results indicate implementation of a formal policy and application process may have reduced incomplete projects. This study examined both completed and incomplete projects to identify factors that led to publication. **Discussion:** This study examines the investment by information specialists in KS projects and the characteristics of projects that result in publication. Findings provide insight for more effective collaboration with research teams.

JOURNAL INFORMATION
==============================
NLM Title Abbreviation: J Can Health Libr Assoc
Journal ID: J Can Health Libr Assoc
NLM Journal ID: 101229936
Journal ID: JCHLA

The Journal of the Canadian Health Libraries Association

EISSN: 1708-6892
Publisher: Canadian Health Libraries Association / Association des bibliotèques de la santé du Canada

ARTICLE INFORMATION
==============================
Subjects: CP = Contributed Paper

CP1. A scoping review case study of citation searching indexes and tools

Pawliuk Colleen
BC Children's Hospital

JOURNAL INFORMATION
==============================
NLM Title Abbreviation: J Can Health Libr Assoc
Journal ID: J Can Health Libr Assoc
NLM Journal ID: 101229936
Journal ID: JCHLA

The Journal of the Canadian Health Libraries Association

EISSN: 1708-6892
Publisher: Canadian Health Libraries Association / Association des bibliotèques de la santé du Canada

ARTICLE INFORMATION
==============================
Subjects: CP = Contributed Paper

CP2. A scoping review of automated indexing in Medline -- how did we get here?

Amar-Zifkin Alexandre 1
Chen Eileen 2
Kung Janice Y 3
Giustini Dean 4
1 Université de Montréal,
2 University of California,
3 University of Alberta,
4 University of British Columbia

JOURNAL INFORMATION
==============================
NLM Title Abbreviation: J Can Health Libr Assoc
Journal ID: J Can Health Libr Assoc
NLM Journal ID: 101229936
Journal ID: JCHLA

The Journal of the Canadian Health Libraries Association

EISSN: 1708-6892
Publisher: Canadian Health Libraries Association / Association des bibliotèques de la santé du Canada

ARTICLE INFORMATION
==============================
Subjects: CP = Contributed Paper

CP3. AI research assistants and Cochrane reviews: a comparative analysis of scientific summaries

Boden Catherine
Langman Erin
University of Saskatchewan

JOURNAL INFORMATION
==============================
NLM Title Abbreviation: J Can Health Libr Assoc
Journal ID: J Can Health Libr Assoc
NLM Journal ID: 101229936
Journal ID: JCHLA

The Journal of the Canadian Health Libraries Association

EISSN: 1708-6892
Publisher: Canadian Health Libraries Association / Association des bibliotèques de la santé du Canada

ARTICLE INFORMATION
==============================
Subjects: CP = Contributed Paper

CP4. Anything you can do, AI can do better... or can it? Comparing ChatGPT's search strategy outputs with Cochrane review searches

Howell Katherine
Carlson Rebecca
Jones Emily
Moreton Elizabeth
University of North Carolina

JOURNAL INFORMATION
==============================
NLM Title Abbreviation: J Can Health Libr Assoc
Journal ID: J Can Health Libr Assoc
NLM Journal ID: 101229936
Journal ID: JCHLA

The Journal of the Canadian Health Libraries Association

EISSN: 1708-6892
Publisher: Canadian Health Libraries Association / Association des bibliotèques de la santé du Canada

ARTICLE INFORMATION
==============================
Subjects: CP = Contributed Paper

CP5. Artificial intelligence-supported screening: does it impact evidence certainty and time to complete a rapid review?

Neil-Sztramko Sarah 1 2
Traynor Robyn 2
Clark Emily 2
Toomey Elaine 3
Dobbins Maureen 1 2
1 McMaster University,
2 National Collaborating Centre for Methods and Tools,
3 University of Galway

JOURNAL INFORMATION
==============================
NLM Title Abbreviation: J Can Health Libr Assoc
Journal ID: J Can Health Libr Assoc
NLM Journal ID: 101229936
Journal ID: JCHLA

The Journal of the Canadian Health Libraries Association

EISSN: 1708-6892
Publisher: Canadian Health Libraries Association / Association des bibliotèques de la santé du Canada

ARTICLE INFORMATION
==============================
Subjects: CP = Contributed Paper

CP6. Assessing the Canadian digital health landscape: opportunities for improved data sharing and research

Foote Alyssa
World Data System

JOURNAL INFORMATION
==============================
NLM Title Abbreviation: J Can Health Libr Assoc
Journal ID: J Can Health Libr Assoc
NLM Journal ID: 101229936
Journal ID: JCHLA

The Journal of the Canadian Health Libraries Association

EISSN: 1708-6892
Publisher: Canadian Health Libraries Association / Association des bibliotèques de la santé du Canada

ARTICLE INFORMATION
==============================
Subjects: CP = Contributed Paper

CP7. Critiquing systematic review search methods in the Cass Review: developing a comprehensive checklist

Sanders Elizabeth 1
Yates Elizabeth 2
1 Lamar University,
2 Brock University

JOURNAL INFORMATION
==============================
NLM Title Abbreviation: J Can Health Libr Assoc
Journal ID: J Can Health Libr Assoc
NLM Journal ID: 101229936
Journal ID: JCHLA

The Journal of the Canadian Health Libraries Association

EISSN: 1708-6892
Publisher: Canadian Health Libraries Association / Association des bibliotèques de la santé du Canada

ARTICLE INFORMATION
==============================
Subjects: CP = Contributed Paper

CP8. Developing a scaffolded learning framework for librarians new to health sciences

Werner Debra A. 1
Kamarei Zahra 2
Rethlefsen Melissa L. 3
Helwig Melissa 4
1 University of Chicago,
2 Arkansas Colleges of Health Education,
3 University of New Mexico,
4 Toronto Metropolitan University

JOURNAL INFORMATION
==============================
NLM Title Abbreviation: J Can Health Libr Assoc
Journal ID: J Can Health Libr Assoc
NLM Journal ID: 101229936
Journal ID: JCHLA

The Journal of the Canadian Health Libraries Association

EISSN: 1708-6892
Publisher: Canadian Health Libraries Association / Association des bibliotèques de la santé du Canada

ARTICLE INFORMATION
==============================
Subjects: CP = Contributed Paper

CP9. Evaluating the performance of broad and narrow search strategies when using machine learning-based software for title/abstract screening

Swab Michelle
Memorial University

JOURNAL INFORMATION
==============================
NLM Title Abbreviation: J Can Health Libr Assoc
Journal ID: J Can Health Libr Assoc
NLM Journal ID: 101229936
Journal ID: JCHLA

The Journal of the Canadian Health Libraries Association

EISSN: 1708-6892
Publisher: Canadian Health Libraries Association / Association des bibliotèques de la santé du Canada

ARTICLE INFORMATION
==============================
Subjects: CP = Contributed Paper

CP10. Investigating the impact of the NLM automatic indexer on information retrieval using citation metadata

Garlock Emma S 1
Bartlett Joan C 2
1 University of Ottawa,
2 McGill University

JOURNAL INFORMATION
==============================
NLM Title Abbreviation: J Can Health Libr Assoc
Journal ID: J Can Health Libr Assoc
NLM Journal ID: 101229936
Journal ID: JCHLA

The Journal of the Canadian Health Libraries Association

EISSN: 1708-6892
Publisher: Canadian Health Libraries Association / Association des bibliotèques de la santé du Canada

ARTICLE INFORMATION
==============================
Subjects: CP = Contributed Paper

CP11. Invisible in the index: how Medline indexing excludes intersex people

Philippopoulos Eleni
McGill University

JOURNAL INFORMATION
==============================
NLM Title Abbreviation: J Can Health Libr Assoc
Journal ID: J Can Health Libr Assoc
NLM Journal ID: 101229936
Journal ID: JCHLA

The Journal of the Canadian Health Libraries Association

EISSN: 1708-6892
Publisher: Canadian Health Libraries Association / Association des bibliotèques de la santé du Canada

ARTICLE INFORMATION
==============================
Subjects: CP = Contributed Paper

CP12. Making the case for play in your library

Bradley-Ridout Glyneva
University of Toronto

JOURNAL INFORMATION
==============================
NLM Title Abbreviation: J Can Health Libr Assoc
Journal ID: J Can Health Libr Assoc
NLM Journal ID: 101229936
Journal ID: JCHLA

The Journal of the Canadian Health Libraries Association

EISSN: 1708-6892
Publisher: Canadian Health Libraries Association / Association des bibliotèques de la santé du Canada

ARTICLE INFORMATION
==============================
Subjects: CP = Contributed Paper

CP13. Optimizing communication and data collection for a systematic review team using Microsoft Power Automate®

Jones Emily
Baldwin-SoRelle Carrie
Carlson Rebecca
University of North Carolina

JOURNAL INFORMATION
==============================
NLM Title Abbreviation: J Can Health Libr Assoc
Journal ID: J Can Health Libr Assoc
NLM Journal ID: 101229936
Journal ID: JCHLA

The Journal of the Canadian Health Libraries Association

EISSN: 1708-6892
Publisher: Canadian Health Libraries Association / Association des bibliotèques de la santé du Canada

ARTICLE INFORMATION
==============================
Subjects: CP = Contributed Paper

CP14. Promoting the use of synthesized evidence in public health decision making: a rapid evidence service and repository of public health evidence syntheses

Neil-Sztramko Sarah 1 2
Miller Alanna 2
Traynor Robyn 2
Caldwell Sophia 2
Camargo Krishian 2
Clark ZEmily 2
Rogers Kristin 2
Dobbins Maureen 1 2
1 McMaster University,
2 National Collaborating Centre for Methods and Tools

JOURNAL INFORMATION
==============================
NLM Title Abbreviation: J Can Health Libr Assoc
Journal ID: J Can Health Libr Assoc
NLM Journal ID: 101229936
Journal ID: JCHLA

The Journal of the Canadian Health Libraries Association

EISSN: 1708-6892
Publisher: Canadian Health Libraries Association / Association des bibliotèques de la santé du Canada

ARTICLE INFORMATION
==============================
Subjects: CP = Contributed Paper

CP15. Rooted in competence: using the MLA professional competencies to redesign a health information course

Pawliuk Colleen
BC Children's Hospital

JOURNAL INFORMATION
==============================
NLM Title Abbreviation: J Can Health Libr Assoc
Journal ID: J Can Health Libr Assoc
NLM Journal ID: 101229936
Journal ID: JCHLA

The Journal of the Canadian Health Libraries Association

EISSN: 1708-6892
Publisher: Canadian Health Libraries Association / Association des bibliotèques de la santé du Canada

ARTICLE INFORMATION
==============================
Subjects: CP = Contributed Paper

CP16. Search strategies as research data: new perspectives on documentation and sharing practices

Cunningham Heather 1
Martyniuk Julia 1
Boruff Jill 2
Calleja Sabine 2
Rod Alisa 2
Orchanian-Cheff Ani 3
Pincivy Alix 4
Ziegler Daniela 5
Gunson Karly 1
1 University of Toronto,
2 McGill University,
3 University Health Network,
4 Centre Hospitalier Universitaire Sainte-Justine,
5 Université de Montréal

JOURNAL INFORMATION
==============================
NLM Title Abbreviation: J Can Health Libr Assoc
Journal ID: J Can Health Libr Assoc
NLM Journal ID: 101229936
Journal ID: JCHLA

The Journal of the Canadian Health Libraries Association

EISSN: 1708-6892
Publisher: Canadian Health Libraries Association / Association des bibliotèques de la santé du Canada

ARTICLE INFORMATION
==============================
Subjects: CP = Contributed Paper

CP17. Stronger together: adventures in instruction and resource sharing to cultivate aspiring nursing students

Antunez Marilia 1
Taylor Christa 2
1 University of Akron,
2 Barberton High School

JOURNAL INFORMATION
==============================
NLM Title Abbreviation: J Can Health Libr Assoc
Journal ID: J Can Health Libr Assoc
NLM Journal ID: 101229936
Journal ID: JCHLA

The Journal of the Canadian Health Libraries Association

EISSN: 1708-6892
Publisher: Canadian Health Libraries Association / Association des bibliotèques de la santé du Canada

ARTICLE INFORMATION
==============================
Subjects: CP = Contributed Paper

CP18. The Book Nook: creating a leisure reading space for staff and learners at three hospital library sites

Farrell Ashley
Glaizer Bianna
Chui Cynthia
Fazelzad Rouhi
University Health Network

JOURNAL INFORMATION
==============================
NLM Title Abbreviation: J Can Health Libr Assoc
Journal ID: J Can Health Libr Assoc
NLM Journal ID: 101229936
Journal ID: JCHLA

The Journal of the Canadian Health Libraries Association

EISSN: 1708-6892
Publisher: Canadian Health Libraries Association / Association des bibliotèques de la santé du Canada

ARTICLE INFORMATION
==============================
Subjects: CP = Contributed Paper

CP19. The development of a freely available module series introducing researchers to all stages of the systematic review process

McKeown Sandra 1
Ritonja Jennifer 2 3
Soleas Eleftherios 1
1 Queen's University,
2 McGill University,
3 St. Mary's Research Centre

JOURNAL INFORMATION
==============================
NLM Title Abbreviation: J Can Health Libr Assoc
Journal ID: J Can Health Libr Assoc
NLM Journal ID: 101229936
Journal ID: JCHLA

The Journal of the Canadian Health Libraries Association

EISSN: 1708-6892
Publisher: Canadian Health Libraries Association / Association des bibliotèques de la santé du Canada

ARTICLE INFORMATION
==============================
Subjects: CP = Contributed Paper

CP20. The involvement of librarians and library technicians in knowledge syntheses published by researchers from Quebec universities: an overview

Clar Monique 1
Courcelles Michel 2
1 Université de Montréal,
2 INRS Armand-Frappier Santé Biotechnologie Research Centre

JOURNAL INFORMATION
==============================
NLM Title Abbreviation: J Can Health Libr Assoc
Journal ID: J Can Health Libr Assoc
NLM Journal ID: 101229936
Journal ID: JCHLA

The Journal of the Canadian Health Libraries Association

EISSN: 1708-6892
Publisher: Canadian Health Libraries Association / Association des bibliotèques de la santé du Canada

ARTICLE INFORMATION
==============================
Subjects: CP = Contributed Paper

CP21. Where did the reviews go?: the good, the bad, and the unfinished

Osborne Zachary 1 2
Ziegler Carolyn 1
Kishibe Teruko 1
Boghosian Talin 1
1 Unity Health Toronto,
2 Seneca Polytechnic

